# The light-makeup advantage in facial processing: Evidence from event-related potentials

**DOI:** 10.1371/journal.pone.0172489

**Published:** 2017-02-24

**Authors:** Keiko Tagai, Hitomi Shimakura, Hiroko Isobe, Hiroshi Nittono

**Affiliations:** 1 Shiseido Global Innovation Center, Kanagawa, Japan; 2 Graduate School of Human Sciences, Osaka University, Suita, Japan; University of Hong Kong, HONG KONG

## Abstract

The effects of makeup on attractiveness have been evaluated using mainly subjective measures. In this study, event-related brain potentials (ERPs) were recorded from a total of 45 Japanese women (*n* = 23 and *n* = 22 for Experiment 1 and 2, respectively) to examine the neural processing of faces with no makeup, light makeup, and heavy makeup. To have the participants look at each face carefully, an identity judgement task was used: they were asked to judge whether the two faces presented in succession were of the same person or not. The ERP waveforms in response to the first faces were analyzed. In two experiments with different stimulus probabilities, the amplitudes of N170 and vertex positive potential (VPP) were smaller for faces with light makeup than for faces with heavy makeup or no makeup. The P1 amplitude did not differ between facial types. In a subsequent rating phase, faces with light makeup were rated as more attractive than faces with heavy makeup and no makeup. The results suggest that the processing fluency of faces with light makeup is one of the reasons why light makeup is preferred to heavy makeup and no makeup in daily life.

## Introduction

Facial makeup, which is intended to accentuate one’s attractiveness to others, might be crucial for many women in their daily lives [1,2]. When they wear makeup, women are usually interested in what type of makeup creates the most attractiveness and is most appealing. Women wear various kinds of makeup to look pretty, glamorous, or “cool” for purposes of self-identity and self-expression, which gives a certain impression to others depending on the social situation or context [3].

For women, makeup manipulates the typical femininity of the face by modifying the bilaterally symmetric balance, the configurations of parts of the eyes and mouth, and skin color and texture [4]. Women wear light or heavy makeup depending on the purpose and setting. Wearing light makeup is suitable for many daily situations, while wearing heavy makeup fits into events like a ceremony or party. Here we use the terms light makeup and heavy makeup as relative terms. The difference is the amount of manipulation to the face [5]. Light makeup tends to give the impression of natural femininity and softness to others. For example, a natural ruddy and glossy color and texture are applied to the facial skin and lips, while the eyebrows are drawn in gentle curves, and eye shadow is blurred into the skin tone. The soft redness and lightness of the skin and the lips are known to enhance femininity and the appearance health [6, 7]. Whereas light makeup is based on natural facial features, heavy makeup is clearly perceivable and tends to give an impression of glamorousness and coolness to others [8]. For example, the skin is made up matte, while dark colors are applied to the eyes and lips and straight lines are drawn to emphasize the contrast of the whole face [3, 9].

The effects of makeup on attractiveness have been evaluated using mainly subjective measures. For example, Mulhern et al. (2003) reported that both men and women judged Caucasian female faces with full makeup more attractive than faces with lip-only makeup or no makeup [9]. Nash et al. (2006) found that the same women's facial photographs were perceived as healthier, more confident, with a greater earning potential, and with more prestigious jobs when they were presented wearing cosmetics than when presented without [10]. Etcoff et al. (2011) revealed that there were differences in judgments of likability and trustworthiness between natural (light) makeup and glamorous (heavy) makeup [8]. The light makeup was suggested to convey a sense of social cooperation. Recently, Tagai et al. (2016) found that faces with light makeup were remembered more correctly and rated more attractive than faces with heavy makeup [11]. The highest recognition performance was obtained for faces with no makeup, because faces with no makeup have idiosyncratic features that make individual faces distinctive from the others. Light makeup involves a more natural application of cosmetics, and the distinctiveness of facial features is more retained relative to heavy makeup. Therefore, faces with light makeup were remembered more correctly than faces with heavy makeup. However, the underlying process of how faces wearing cosmetics, especially with different types of makeup, are processed in the brain remains unclear.

The purpose of this study is to illustrate the effect of makeup on interpersonal cognition by examining event-related brain potential (ERP) responses to faces with no makeup, light makeup, and heavy makeup. ERP measures have been used to examine the differences in processing between attractive and unattractive faces [12–15]. To our knowledge, there is no published ERP study about the effects of makeup on facial processing. However, because the purpose of makeup is to make a face more attractive, the effects of makeup can be predicted from the known effects of facial attractiveness on ERPs. Trujillo et al. (2013) found that the amplitudes of the N170 component were smaller for attractive faces than for unattractive faces [12]. The reduction of N170 amplitude was also observed for mathematically averaged faces, which were also rated to be highly attractive [13]. Trujillo et al. suggested that a smaller N170 reflected the engagement of fewer neural resources and that attractive faces were processed more fluently in the early stages of facial processing, possibly because the attractive faces were closer to the prototype of human faces [12]. Consistent with this hypothesis, Trujillo et al. found that chimpanzee’s faces elicited a larger N170 than did human faces. Other studies have also reported that attractive faces are associated with a smaller N170 amplitude. Halit et al. (2000) found that N170 amplitude was smaller for attractive faces than for unattractive faces of adult women [14]. Hahn et al. (2015) used faces of babies and adults and found that more attractive faces elicited a smaller N170 than less attractive faces, regardless of the model’s age, while babies’ faces generally elicited a larger N170 than did adults’ faces [15].

Another line of research suggests that unnatural faces elicited a larger N170 response. Minami et al. (2011) reported that N170 amplitude was larger and N170 latency was longer for a bluish-colored (thus, atypical and irregular) face than for a natural-colored face [16]. Using face stimuli with 8 different colors generated by rotating the color distribution of the original face image around the white point by 45 degrees, Nakajima et al. (2012) showed that N170 amplitude in the left posterior temporal site increased for an unnaturally colored face in proportion to the increase in hue angle from the naturally colored face [17]. They suggested that N170 amplitude in the left hemisphere reflected the processing of facial color information during an early stage of facial detection. Schulz et al. (2012) showed that N170 amplitude in the right hemisphere was greater for a spatially exaggerated face than for veridical and non-exaggerated faces containing mostly typical shapes similar to the average [18].

On the other hand, Halit et al. (2000) reported no N170 differences between the original faces and the unnaturally stretched faces of which the eyes were moved upward and the mouth was moved downward [14]. Wiese et al. (2014) reported that attractive and unattractive faces elicited a very similar N170 when the distinctiveness of the faces was matched [19]. The N170 amplitude might be related more closely to distinctiveness rather than attractiveness.

Although there are some exceptions, the previous findings generally suggest that N170 amplitude is modulated by the atypicality or unnaturalness of the face and that fluent processing of a face is associated with a smaller N170 response. As processing fluency leads to positive affect [20, 21], faces that are processed fluently would be rated to be more attractive.

In the present study, we investigated the influence of light makeup and heavy makeup on facial perception by means of ERP and ratings of attractiveness. We hypothesized that makeup influences the early stages of facial processing that are reflected in the N170 amplitude and its polarity-inversed counterpart, the vertex positive potential (VPP). If the N170 amplitude is inversely related to the processing fluency of the face, as proposed by Trujillo et al. (2013), a smaller N170 amplitude would be expected for faces with makeup than for faces with no makeup, because makeup would conceal the negative features of individual faces and make them closer to the average [12]. On the other hand, heavy makeup involves a greater physical manipulation of the natural face than does light makeup so that the impression of the makeup style itself becomes prominent [11]. Therefore, faces with heavy makeup would be perceived to be less natural than faces with light makeup. If this is the case, the N170 amplitude would be larger for faces with heavy makeup than for faces with light makeup. In line with previous studies showing that lighter makeup was preferred in daily situations [11, 22], faces with light makeup would be judged to be more attractive than faces with heavy makeup, which in turn would be more attractive than faces with no makeup.

In addition to the N170, the P1 component, which precedes the N170, was analyzed to see whether any physical differences in the faces and/or attention modulation affect ERP responses to faces with makeup. Halit et al. (2000) showed that the P1 amplitude was larger for unattractive faces than attractive faces [14]. They also showed that atypically stretched faces elicited larger P1 amplitude than did the original faces. They concluded that faces which deviated from a prototypical face elicited larger P1 amplitudes. On the other hand, Zheng et al. (2012) reported that the P1 component did not differ between faces that varied in identity strength as a result of morphing individual faces to an “average” face [23]. The latter result suggests that small physical differences in faces do not affect P1 amplitudes. Considering these findings, we did not expect P1 amplitude differences for no makeup, light makeup, and heavy makeup for faces of the same persons. P1 amplitude is also known to increase for emotionally charged faces (e.g., fearful faces) through attentional modulation [24]. However, this is not applicable to the present study as we only used neutral faces.

To have the participants look at each face carefully, an identity judgment task similar to that in Ueda and Koyama (2014) was used [25]. In this task, two faces were presented sequentially, and participants were asked to judge whether the two faces were of the same person or not. The ERPs in response to the first face were analyzed. This was because the response to the first face was not contaminated by other cognitive processes such as judgment and response selection, and thus, was considered as a typical situation in which a person carefully looks at a face. Two experiments were conducted using the same stimulus set with minor differences in procedure.

## Method

### Experiment 1

#### Participants

Thirty Japanese women were recruited by a research firm and paid for participation. The conditions of recruitment were (1) physically and mentally unimpaired, (2) being right-handed determined according to self-report, (3) having reported normal or corrected to normal vision determined according to self-report, and (4) wearing makeup with a mid-priced lipstick and foundation three times a week or more. We considered that women who wear makeup regularly in daily life are aware of the effects of makeup and can make use of the advantage it provides. This was also the reason why only female participants were recruited. Participants gave written informed consent before testing. The Ethical Committee of the Shiseido Global Innovation Center approved this study. Participants who showed a large number of eye blinks during electroencephalogram (EEG) recording and who thus had an insufficient number of artifact-free EEG trials were excluded from the analysis (see the Electrophysiological recording section below for the criteria). The following analysis was conducted on the data from 23 women (mean age = 29.0 years old, *SD* = 7.0, range = 20–33).

#### Stimuli

**Fig 1:**
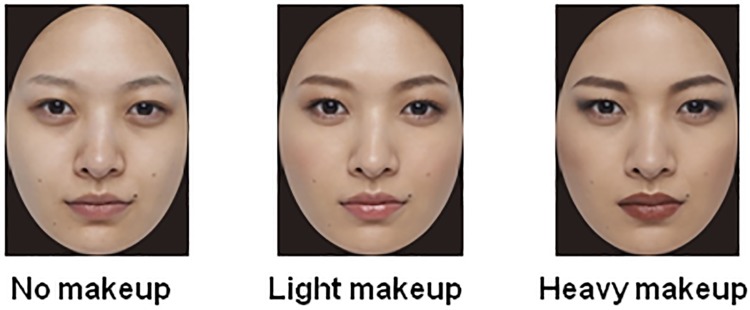
Examples of facial images (no makeup, light makeup, and heavy makeup).

The stimuli were 18 Japanese female faces that encompassed three types of facial images (no makeup, light makeup, and heavy makeup) of six female models (25–35 years old). All the styles of makeup were manipulated by a professional makeup artist. Fig 1 shows some examples. A professional photographer took photos of the female models for each makeup category. The photos for the light makeup and heavy makeup categories were then carefully adjusted by the photographer by adding a slight appearance of gloss to the lips for the light makeup category and darkening the area around the eye for the heavy makeup category. The styles of makeup were similar to those in Tagai et al. (2016) [11]. For the light makeup, the foundation base was blended smoothly into the skin tone. The outlines of the facial features were blurred, and the shapes of the eyebrows were smoothed to create softness. The eye shadow was also blurred to blend into the skin tone. The lips were tinged with a reddish gloss. A soft red was used on the cheeks and blended from the center into the skin at the periphery. For the heavy makeup, a matte foundation covered the skin completely. The outlines of the facial features were emphasized with straight lines. The eyebrows were drawn in a dark color with sharp, straight lines to make the center of the brow higher. Dark eye shadow was applied to produce long-slitted eyes. A matte brownish color with a sharp outline was added to the lips. The cheek color was a dark red-brown and blurred to accent the cheekbone. The quality of all the pictures, including faces with no makeup, was checked carefully by a professional makeup artist and a makeup product manager to meet the standards of cosmetic quality and naturalness. The size of the original pictures was 2771 × 4153 pixels (360 dpi), and the pictures were edited into a 368 × 500-pixel size. An oval occluding window of the same size was applied to each photograph to hide the background and hair. There were no significant differences in brightness (calculated using Photoshop Ver. 10.0) among the three types of faces of six models, *F*(2, 4) = 2.30, *p* = .217, η_p_^2^ = .54.

#### Procedure

Participants were seated in a comfortable chair in a dimly lit room. The stimuli were presented on a computer screen (VIEWPixx, VPixx Technologies, Quebec, Canada) using Inquisit 4.0 (Millisecond software, Seattle, WA). All the faces were displayed against a black background and subtended 8° in width and 10° in height at a viewing distance of 70 cm.

**Fig 2:**
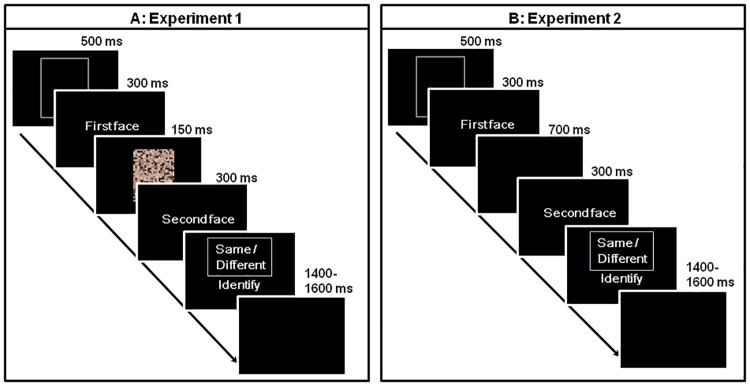
A schematic representation of the identity judgment task used in Experiments 1 and 2.

Fig 2 shows a schematic diagram of the identity judgment task. In Experiment 1, the protocol used in Ueda and Koyama (2014) [25] was replicated. Two faces were presented in sequence: after a white frame was presented for 500 ms, the first face appeared for 300 ms, followed by a visual mask for 150 ms, and then the second face appeared for 300 ms. Participants were asked to judge whether the two faces were of the same person or not, and they responded by pressing one of two buttons on a response pad (RB-530, Cedrus, CA). The right or left hand for the response was counterbalanced across participants. The next trial began 1,400–1,600 ms after the button press.

The faces were changed either from no makeup to makeup (*makeup* sequence) or from makeup to no makeup (*no-makeup* sequence). Each sequence consisted of 144 trials. The first face and the second face were of the same person in 72 trials and of different persons in 72 trials. The faces of the six models were presented equiprobably. In the makeup sequence, the first face was always a no-makeup face and the second face was one of the three types (no makeup, light makeup, or heavy makeup, *p* = .33 each). In the no-makeup sequence, the first face was one of the three types and the second face was always a face with no makeup. Table 1 shows the full combinations of the S1 and S2. The makeup and no-makeup sequences were presented alternately in blocks, each having 72 trials. The total number of trials was 288. ERPs in response to the first faces were recorded irrespective of the same or different pairs, because the same-different distinction did not exist until the second face appeared. The trials were presented in a random order. The participants were given a short break after every 36 trials. Before starting the experiment, participants ran through six trials using three sample faces that were not used in the experiment.

**Table 1:** Summary of the response accuracy in the identity judgement task.

	Condition				MANOVA		
Experiment 1	S1	S2	*M*	*SD*	*F*(2, 21)	*p*	η_*p*_^*2*^
	No makeup	No makeup	.92_a_	.05	36.10	<.001	0.77
	No makeup	Light makeup	.84_b_	.07			
	No makeup	Heavy makeup	.82_b_	.07			
	No makeup	No makeup	.93_a_	.05	38.95	<.001	0.79
	Light makeup	No makeup	.84_b_	.09			
	Heavy makeup	No makeup	.81_b_	.07			
Experiment 2	S1	S2	*M*	*SD*	*F*(2, 20)	*p*	η_*p*_^*2*^
	No makeup	No makeup	.91_a_	.06	15.22	<.001	0.60
	No makeup	Light makeup	.84_b_	.07			
	No makeup	Heavy makeup	.82_b_	.07			
	Light makeup	No makeup	.82_b_	.08	12.23	<.001	0.55
	Light makeup	Light makeup	.89_a_	.05			
	Light makeup	Heavy makeup	.84_b_	.07			
	Heavy makeup	No makeup	.81_b_	.08	3.79	.040	0.28
	Heavy makeup	Light makeup	.84_a_	.07			
	Heavy makeup	Heavy makeup	.86_a_	.07			

Note: A one-way multivariable analysis of variance (MANOVA) was conducted separately for the makeup sequence and no-makeup sequence in Experiment 1 and for each S1 makeup type in Experiment 2. Means in the same MANOVA that do not share subscripts differ at *p* <.05.

#### Subjective impressions

After completing the identity judgment task, participants were asked about their subjective impressions of the faces. First, they conducted a forced-choice task. Two makeup types of the same person were presented side by side, and participants were told to intuitively choose the face that gave a better impression without deliberation. In addition to the six models used in the identity judgement task, four virtual persons were created using computer graphics. The quality of these faces was also checked by the makeup artist and the makeup production manager to be comparable with the quality of the real faces with makeup. The light-makeup and heavy-makeup versions of each person’s face were compared. As each version of the faces appeared either in the right position or in the left position, the total number of pairs was 20. The number of trials in which the participant chose the face with light makeup was counted. In addition, as filler items, the same face was presented on both sides in 20 additional trials so that the participants performed the task carefully.

At the end of the experiment, the participants provided subjective ratings of nine dimensions (attractive, feminine, natural, friendly, youthful, pretty, bright, blatant, and cool) using a 100-point visual analog scale on paper for their impressions of the faces of 10 models with no makeup, light makeup, and heavy makeup. Each participant rated only one makeup type of each model, that is, 10 faces in total. The makeup types of each model were counterbalanced across participants.

#### Electrophysiological recording

Electroencephalogram (EEG) data were recorded from 19 scalp sites (Fp1, Fp2, F3, F4, F7, F8, Fz, C3, C4, Cz, P3, P4, Pz, T3, T4, T5, T6, O1, and O2) according to the 10–20 system using BIOSEMI active electrodes (BIOSEMI, Amsterdam, The Netherlands). Horizontal and vertical electrooculograms (EOGs) were recorded from the outer anti of both eyes and from above and below them. The sampling rate was 256 Hz.

The EEG data were converted to the average reference, and an offline filter of 0.1–30 Hz was applied. Ocular artifacts were corrected by the independent component analysis (ICA) function implemented in the Brain Vision Analyzer 2.1.0 (Brain Products, Gilching, Germany). ERPs in response to the first faces were calculated for 400 ms (from 100 ms before and 300 ms after stimulus onset). Because a visual mask was presented 300 ms after stimulus onset, we only analyzed the epoch before the mask that might evoke other ERP responses. The periods containing artifacts exceeding ±80 μV in amplitude (after the ocular correction) were rejected from the average. The participants who had an insufficient number of artifact-free trials (less than 36 trials in Experiment 1 and less than 34 trials in Experiment 2) were excluded from the analysis. Each ERP waveform was aligned to the 100-ms pre-stimulus baseline by subtracting the mean amplitude of this period from each point of the waveform. ERPs were quantified using the mean amplitudes of P1 (80–110 ms) components for O1 and O2 and N170 (120–170 ms) components for T5, T6, and Cz.

#### Statistical analysis

The response accuracy was analyzed by a multivariate analysis of variance (MANOVA) with repeated measures. The same analysis was also conducted on the data after arcsine transformation. Because both analyses yielded similar results, the results without transformation are reported here. The percentage of the choice of faces with light makeup in a forced-choice task was analyzed by a binomial test. Subjective rating scores were analyzed by a MANOVA with a single factor of makeup type (no makeup, light makeup, and heavy makeup). The mean amplitudes for P1 and N170 were analyzed by a MANOVA with two factors: makeup type (no makeup, light makeup, and heavy makeup) and electrode site (O1 and O2 for P1; T5, T6, and Cz for N170). Because the present study used repeated-measures designs, we follow the recommendation of Vasey and Thayer (1987) and report MANOVA solutions to compensate for possible type I error inflation by the violation of sphericity [26]. The response accuracy was analyzed by a MANOVA. The same analysis was also conducted on the data after arcsine transformation. Because both analyses yielded similar results, the results without transformation are reported here. Post-hoc multiple comparisons were made using Shaffer’s (1986) modified sequentially rejective multiple test procedure, which extends Bonferroni *t* tests to a stepwise fashion and increases the probability of detecting differences without changing the control of the Type I error [27]. The significance level was set to .05 for all analyses.

### Experiment 2

Because Experiment 1 replicated the protocol of Ueda and Koyama (2014) [25], the presentation rates of the three types of faces were unequal: *p* = .67, .17, and .17 for faces with no makeup, light makeup, and heavy makeup, respectively. In Experiment 2, we equalized the presentation rates of the three types of faces (*p* = .33). Moreover, to cause participants to think of the identity of the faces deliberately, a visual mask was not presented and the interstimulus interval between the first and second faces was extended to 700 ms. Unless otherwise mentioned, the methods were identical to Experiment 1.

#### Participants

Thirty Japanese women were recruited and participated in Experiment 2. They had not participated in Experiment 1. Based on the same criteria used in Experiment 1, the data of 22 women (mean age = 32.6 years old, *SD* = 2.4, range = 27–35) were analyzed.

#### Procedure

In the identity judgement task, a visual mask was not used and the interval between the offset of the first face and the onset of the second face was extended to 700 ms. In a total of 216 trials, half of the trials were the same pairs, and the other half of the trials were different pairs. Faces with no makeup, light makeup, and heavy makeup were presented 72 times each for the first and second face positions. Table 1 shows the full combinations of S1 and S2. Again, ERPs in response to the first faces were recorded.

#### Subjective impressions

The forced-choice task was modified to include faces with no makeup. The three types of faces of the six real models (18 pictures in total) were used. For each model, three makeup types could appear either in the left or right position, which resulted in nine combinations (no–no, no–light, no–heavy, light–no, light–light, light–heavy, heavy–no, heavy–light, and heavy–heavy). A set of 54 trials (9 combinations × 6 models) was conducted twice. For each combination, the chosen type was coded as +1 and the unchosen type was coded as -1. The sum of the values was calculated for each makeup type. If a certain makeup type was chosen consistently, the sum would be 48. Subjective ratings using analog visual scales were not administered.

## Results

### Experiment 1

#### Response accuracy

Table 1 shows the response accuracy in the identity judgement task. Overall, the performance was good and shows that the participants looked at the faces carefully. A MANOVA was conducted separately for the makeup sequence (i.e., S1 was a face with no makeup) and for the no-makeup sequence (i.e., S2 was a face with no makeup). For both sequences, the response accuracy was higher when both S1 and S2 were faces with no makeup than when S1 and S2 were of different makeup types.

#### Subjective impressions

In the forced-choice task, faces with light makeup were selected as a face with a better impression more frequently than faces with heavy makeup. On average, faces with light makeup were chosen 17.87 times (89.3%) out of 20 pairs (binominal test, *p* = .001, two-tailed).

Table 2 summarizes the mean scores of nine subjective rating scales for the three makeup types along with the statistical results. The ratings of attractiveness and femininity were highest for faces with light makeup; faces with heavy makeup were in the second place, and faces with no makeup were rated to be the least attractive and feminine. The ratings of naturalness were inversely related to the cosmetic manipulation: No makeup was most natural, light makeup was in the second place, and heavy makeup was least natural. No makeup and light makeup were rated as looking more friendly and youthful than heavy makeup. Faces with light makeup were rated as looking prettier than faces with no makeup and heavy makeup, while faces with heavy makeup were rated as looking less pretty than faces with no makeup. Both light and heavy styles of makeup were rated as brighter than no makeup. Heavy makeup was rated as looking more blatant than no makeup and light makeup, while light makeup was rated as looking more blatant than no makeup. Also, heavy makeup was rated as looking cooler than light makeup and no makeup. The numerical details of the statistics are shown in Table 1.

**Table 2:** Summary of the subjective ratings for faces with no makeup, light makeup, and heavy makeup.

	Makeup type								
	No makeup		Light makeup		Heavy makeup				
	*M*	*SD*	*M*	*SD*	*M*	*SD*	*F*(2, 21)	*p*	η_*p*_^*2*^
*Attractive*	26.53_c_	16.83	54.97_a_	15.11	40.84_b_	17.17	21.94	<.001	.68
*Feminine*	38.86_c_	17.52	67.20_a_	14.51	55.33_b_	19.54	15.99	<.001	.60
*Natural*	72.05_a_	20.57	46.47_b_	18.44	18.57_c_	12.16	55.82	<.001	.84
*Friendly*	50.90_a_	16.75	56.23_a_	15.46	30.63_b_	13.43	28.39	<.001	.73
*Youthful*	39.99_a_	21.58	47.79_a_	21.46	22.78_b_	14.10	19.74	<.001	.65
*Pretty*	31.61_b_	18.83	49.66_a_	18.62	21.00_c_	13.40	21.97	<.001	.68
*Bright*	22.37_b_	14.90	61.02_a_	12.46	53.34_a_	19.64	36.45	<.001	.78
*Blatant*	14.72_c_	11.41	46.60_b_	17.29	61.15_a_	18.00	68.42	<.001	.87
*Cool*	32.56_b_	17.23	33.83_b_	16.07	54.19_a_	17.30	12.51	<.001	.54

Note: Means in the same row that do not share subscripts differ at *p* <.05.

#### P1 and N170

**Fig 3:**
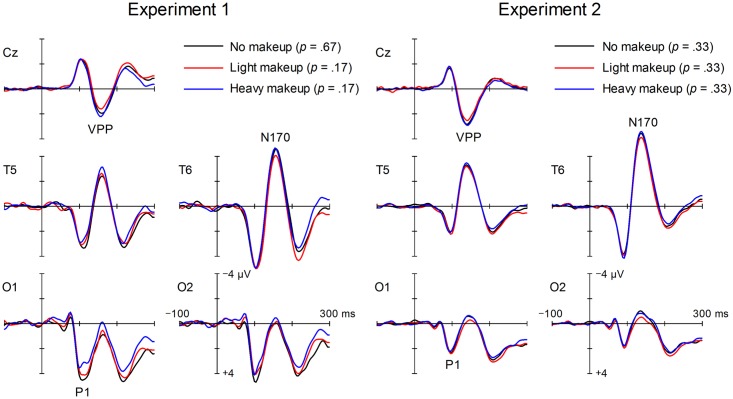
Grand mean ERP waveforms elicited by faces with no makeup, light makeup, and heavy makeup. Frequencies of appearance are shown in parentheses. VPP: vertex positive potential.

Fig 3 illustrates the grand mean ERP waveforms elicited by faces with no makeup, light makeup, and heavy makeup. All types of faces elicited a positive wave (P1) peaking around 100 ms and a negative wave (N170) peaking around 170 ms after stimulus onset. Table 3 shows the mean amplitude values (μV) of P1 and N170 in response to the three types of faces along with the statistical results. There were marginally significant differences in P1 amplitude for no makeup, light makeup, and heavy makeup, Makeup type: *F*(2, 21) = 3.08, *p* = .067, η_*p*_^*2*^ = .23. Multiple comparisons showed that the P1 amplitude was marginally smaller for heavy makeup than for no makeup (*p* = .070). The effect of site and the interaction effect were not significant, *F*(1, 22) = 1.99, *p* = .172, η_p_^2^ = .08; *F*(2, 21) = 0.75, *p* = .486, η_p_^2^ = .07, respectively. On the other hand, the N170 amplitude showed a significant main effect of site and a significant interaction, Makeup type: *F*(2, 21) = 1.63, *p* = .220, η_p_^2^ = .13, Site: *F*(1, 22) = 3.75, *p* = .041, η_p_^2^ = .26, Makeup type × Site: *F*(2, 21) = 3.70, *p* = .022, η_p_^2^ = .44. The significant effect of site reflects the polarity inversion of the N170/VPP. A one-way MANOVA was conducted for each site. The effect of makeup type was significant at T5 and Cz, *F*(2, 21) = 4.05 and 5.21, *p* = .032 and .015, η_p_^2^ = .28 and .33, respectively. Multiple comparisons of means showed that the N170 amplitude was smaller (less negative) for no makeup than for heavy makeup at T5 (*p* = .009) and that its counterpart VPP was smaller (less positive) for light makeup than for heavy makeup at Cz (*p* = .004). The main effect of makeup type was marginally significant at T6, *F*(2, 21) = 2.62, *p* = .097, η_p_^2^ = .20. Multiple comparisons showed that the N170 amplitude was smaller for light makeup than for heavy makeup (*p* = .037). There were no significant differences between no makeup and light makeup in the N170/VPP amplitude and between no makeup and heavy makeup in the VPP amplitude. In sum, faces with heavy makeup elicited a larger N170/VPP than did faces with no makeup or light makeup.

**Table 3:** Mean amplitude values (μV) of P1 and N170/vertex positive potential in response to the three types of faces in Experiment 1 and Experiment 2.

Component	Experiment	Site	Makeup type					
			No makeup		Light makeup		Heavy makeup	
			*M*	*SD*	*M*	*SD*	*M*	*SD*
P1 (80–110 ms)	1	O1	2.54	2.98	2.16	3.18	2.07	2.67
		O2	3.15	3.49	2.51	3.97	2.59	3.68
	2	O1	1.81	1.79	1.92	1.82	1.83	1.83
		O2	1.31	1.95	1.43	2.17	1.25	2.05
N170 (120–170 ms)	1	T5	-0.71_a_	2.68	-0.97	2.43	-1.32_b_	2.37
		T6	-2.42	4.39	-2.00_a_	4.24	-2.69_b_	4.69
		Cz	0.97	1.51	0.66_a_	1.59	1.21_b_	1.64
	2	T5	-2.50	3.14	-2.41	3.05	-2.63	3.37
		T6	-4.36	3.27	-4.10_a_	3.39	-4.57_b_	3.43
		Cz	2.22_b_	1.93	1.97_a_	1.89	2.33_b_	2.14

Note: Means in the same row that do not share subscripts differ at *p* <.05.

### Experiment 2

#### Response accuracy

Table 1 shows the response accuracy for the identity judgement task. Again, the performance was good and showed that the participants looked at the faces carefully. A MANOVA was conducted separately for each S1 makeup type. For all S1 types, identification performance differed significantly between S2 makeup types. The response accuracy was higher when S1 and S2 were of the same makeup type than when S1 and S2 were of different makeup types. Moreover, when S1 and S2 were of the same makeup type, heavy-makeup pairs resulted in the worst performance (*M* = .86) as compared with light-makeup pairs (*M* = .89) and no-makeup pairs (*M* = .91), *F*(2, 20) = 9.12, *p* < .001, η_p_^2^ = .48. Post-hoc pairwise comparisons showed significant differences between heavy makeup and light makeup (*p* = .002) and between heavy makeup and no makeup (*p* = .001).

#### Subjective impressions

In the forced-choice task, faces with light makeup were chosen as faces with a better impression most frequently. Faces with heavy makeup were in second place, and faces with no makeup were the least chosen. The choice measure (varies from 48 to -48) were *M* = 35.91 (*SD* = 10.57), *M* = -7.82 (*SD* = 14.29), and *M* = -28.09 (*SD* = 17.88) for faces with light makeup, heavy makeup, and no makeup, respectively. A Friedman test showed a significant effect of makeup type (χ^2^ = 31.73, df = 2, *p* < .01). Post-hoc multiple comparisons using Wilcoxon signed-rank tests revealed that the measures of the three makeup types differed from one another (*p* < .01).

#### P1 and N170

Fig 3 illustrates the grand mean ERP waveforms elicited by faces with no makeup, light makeup, and heavy makeup. Table 3 shows the mean amplitude values (μV) of P1 and N170 in response to the three types of faces along with the statistical results. No significant differences were found in the mean amplitude values of P1, Makeup type: *F*(2, 20) = 0.39, *p* = .685, η_p_^2^ = .04; Site: *F*(1, 21) = 2.80, *p* = .109, η_p_^2^ = .12; Makeup type × Site: *F*(2, 20) = 0.33, *p* = .723, η_p_^2^ = .03. For the N170 amplitude, the main effects of site and interaction were significant, Makeup type: *F*(2, 20) = 1.38, *p* = .274, η_p_^2^ = .12; Site: *F*(1, 21) = 18.99, *p* < .001, η_p_^2^ = .66; Makeup type × Site: *F*(2, 20) = 3.33, *p* = .033, η_p_^2^ = .43. The significant effect of site reflects the polarity inversion of the N170/VPP. A one-way MANOVA was conducted for each site. The effect of makeup type was significant at T6 and Cz, *F*(2, 20) = 4.13 and 6.30, *p* = .031 and .008, η_p_^2^ = 0.29 and .008, respectively. Multiple comparisons of means showed that the N170 amplitude was smaller (less negative) for light makeup than for heavy makeup at T6 (*p* = .009) and that its counterpart VPP was smaller (less positive) for light makeup than for heavy makeup at Cz (*p* = .002). In addition, the VPP was smaller for light makeup than for no makeup at Cz (*p* = .019). The main effect of makeup type was not significant at T5, *F*(2, 20) = 1.24, *p* = .311, η_p_^2^ = 0.11. Multiple comparisons did not show any amplitude differences between makeup types.

## Discussion

This study examined how makeup influences the early components of ERPs (P1 and N170) in the facial recognition process using faces of the same female models with no makeup, light makeup, and heavy makeup. Two experiments with different stimulus probabilities consistently showed that the amplitude of P1 (80–110 ms) at the occipital sites showed no significant difference between makeup types. In both experiments, the amplitude of N170/VPP (120–170 ms) was smaller for faces with light makeup than for faces with heavy makeup at the central site (Cz). The latter difference was also significant at the right posterior temporal site (T6) in Experiment 2, in which the three types of faces were presented equiprobably. In addition, the N170/VPP amplitude at Cz was smaller for faces with light makeup than for faces with no makeup in Experiment 2. As compared to no makeup and light makeup, heavy makeup deteriorated the performance in the identity judgement task in Experiment 2. In both experiments, faces with light makeup were chosen more frequently as a face with a better impression than faces with heavy makeup. On the other hand, Experiment 2 showed that faces with heavy makeup were chosen more frequently than faces with no makeup.

The result that the P1 amplitude did not significantly differ between makeup types suggests that the ERP differences occurred at a level higher than physical characteristics. Although the P1 amplitude was numerically smaller for heavy makeup than for no makeup in Experiment 1, this difference was not found in Experiment 2. In Experiment 1, differences in stimulus probability across makeup types may affect P1 amplitudes. Therefore, the present study did not provide strong evidence that makeup types affect P1 amplitudes. It has been suggested that the P1 effect depends on low-level visual cues of faces [28, 29], whereas the N170 reflects the configuration analysis of whole faces [30]. Makeup increases facial attractiveness by concealing the negative features of an individual face [4, 8]. As this procedure is similar to that of reducing idiosyncrasies and making the face closer to a prototype, faces with makeup are judged to be more attractive than faces with no makeup. The finding that faces with makeup elicited a smaller N170 response is consistent with the result of Trujillo et al. (2013) [12]. Processing fluency, which was reflected in a smaller N170 amplitude, is argued to be related to positive evaluation [20, 21]. Another possible explanation is that makeup alters the texture and color of the face and creates a smoother appearance for the skin. In this regard, Wiese et al. (2008) reported that the amplitudes of N170 for old faces were larger than for young faces [31] and suggested that the facial details present in old faces, such as wrinkles, might require the processing of high spatial frequencies. They posit that the N170 could reflect the enhanced analysis of these facial components. Komes et al. (2014) found that low-pass filtered blurred faces elicited a smaller N170 [32]. Therefore, the N170 amplitude reduction in the current study may be a result of the textural change effected by makeup.

A puzzling finding is that a smaller N170 amplitude was found for faces with light makeup but not for faces with heavy makeup. With heavy makeup, individual faces become less distinctive and more difficult to be recognized correctly [11]. In a sense, all the faces become closer to a prototype, that is, the style of the makeup. The reason why the N170 amplitude was not reduced for faces with heavy makeup seems to be because the style of the heavy makeup was deviated from the general prototype of natural human faces. This explanation is consistent with the result of subjective ratings: heavy makeup (*M* = 18.57 out of 100) was rated to be significantly less natural than light makeup (*M* = 46.47) and no makeup (*M* = 72.05). In the present study, the N170 amplitude at the right posterior temporal site (T6) was smaller for light makeup than for heavy makeup. Schultz et al. (2012) reported that the N170 amplitude in the right hemisphere was greater for a spatially exaggerated face than for veridical and non-exaggerated faces [18]. Because heavy makeup accentuated the eyes, mouth, cheeks, and the whole face using dark colors and straight lines, it might affect the configuration of a face and make the face less typical.

In both experiments, there were no significant differences in the P1 and N170 amplitudes between faces with heavy makeup and no makeup. Because faces with heavy makeup were rated to be more attractive than faces with no makeup, processing fluency, which is reflected in the reduction of the N170 amplitude, alone does not explain the subjective ratings of attractiveness. Heavy makeup involved larger physical changes in appearance than light makeup. Therefore, the finding that the P1 and N170 amplitudes did not differ between faces with heavy makeup and faces with no makeup supports the idea that the N170 reduction for faces with light makeup (i.e., moderate physical changes) was not due to low-level perceptual differences.

The most accurate facial recognition was achieved when the first and second faces were of the same makeup type (no makeup–no makeup, light makeup–light makeup, or heavy makeup–heavy makeup). This result is reasonable, because the identical pictures were presented twice in half of the trials for these pairs. Interestingly, the identification performance for heavy makeup–heavy makeup pairs was worse than for the other pairs. As Tagai et al. (2016) pointed out, heavy makeup may conceal the idiosyncratic features of the person and thus hinder identity judgement [11]. The light makeup and no makeup did not differ in recognition accuracy. This finding was inconsistent with the result of Ueda and Koyama (2014) [25], who reported that faces wearing light makeup were more easily recognized than those with no makeup. The reason for this discrepancy is unclear. A possible explanation is that the styles of makeup may differ between their study and the present study. However, because Ueda and Koyama (2014) did not describe in detail the characteristics of the light makeup they used [25], we cannot discuss this possibility further.

In the forced-choice task, light makeup was chosen more frequently than heavy makeup as a face with a better impression. This result is consistent with previous findings in Japan [11]. Light makeup was viewed as more attractive and gave more feminine and pretty impressions than both heavy makeup and no makeup, while heavy makeup gave more blatant, cooler, and less natural impressions than light makeup and no makeup. Although this may be due to the effect of recent fashion trends in Japan [3], it is possible that wearing light makeup educes the natural femininity that each woman has individually and shows up her facial characteristics more positively. Jones et al. (2015) argued that people tend to wear makeup heavier than other people deem appropriate, based on a study conducted in the UK [22]. Moreover, Etcoff et al. (2011) showed that light makeup (natural or professional) tends to create a sense of trustworthiness and likability more than heavy (glamorous) makeup, although the ratings of attractiveness were numerically higher for glamorous makeup than for natural makeup [8]. Therefore, wearing light makeup seems to have merit over wearing a heavy makeup in that it will give a better impression to others, if not judged to be most attractive. Examining cultural differences is an interesting topic for future research. In particular, if the N170 response is related to the typicality or closeness to the general prototype of the human face, this early brain potential in response to faces with various types of (or no) makeup might be affected by social and cultural experiences.

There are several limitations in this study. First, we could not determine exactly which features of the light makeup led to the reduction of the N170 response. For example, according to the definition of the present study, the light and heavy styles of makeup used different colors: the light makeup was based on soft red with natural skin tone, whereas the heavy makeup was based on dark brown with matte skin. Minami et al. (2011) and Nakajima et al. (2012) suggested that facial color can affect the N170 amplitude [16, 17]. As makeup is an art, color and shape are not inseparable in actual application. However, the theoretical issue will be resolved by experiments using monotone pictures of faces and spatially filtered, blurred faces. If the N170 reduction for faces with light makeup is due to facial color, the effect would be present for blurred faces but not for monotone faces. If it is due to the blurred and smoothed outlines of facial features, the effect would also be found even when monotone faces are used. Second, we did not find a causal relationship between early ERP responses and attractiveness ratings. In the present study, light makeup was preferred to heavy makeup, and light makeup was associated with a smaller N170 response, which is suggestive of fluent processing. However, for an evening party, for example, heavy makeup may be preferred to light makeup. As facial attractiveness may also be processed differently by task requirement [33], further experiments using different task protocols are required to elucidate the relationship between ERP responses and attractiveness ratings. Third, only female participants were recruited in this study. Cash et al. (1989) reported that made-up faces of American college women were rated as more attractive than their faces without makeup by male peers, but not by female peers [34]. It is worth examining whether men show similar results in the situation where women wear makeup to appeal to the opposite sex.

In summary, the present study suggests that faces with light makeup are processed more fluently than faces with heavy makeup or faces with no makeup. This may be one of the reasons why light makeup is preferred and rated to be more attractive than heavy makeup in daily life. Together with the findings of Tagai et al. (2016) [11], light makeup has an advantage over heavy makeup in that it causes a face to be processed more fluently, gives a better impression, and is remembered more correctly.
